# Digital encounter decision aids linked to clinical practice guidelines: results from user testing SHARE-IT decision aids in primary care

**DOI:** 10.1186/s12911-023-02186-4

**Published:** 2023-05-22

**Authors:** Pieter Van Bostraeten, Bert Aertgeerts, Geertruida Bekkering, Nicolas Delvaux, Anna Haers, Matisse Vanheeswyck, Alexander Vandekendelaere, Niels Van der Auwera, Charlotte Dijckmans, Elise Ostyn, Willem Soontjens, Wout Matthysen, Noémie Schenk, Lien Mertens, Jasmien Jaeken, Thomas Agoritsas, Mieke Vermandere

**Affiliations:** 1 Academic Center for General Practice, department of PH&PC, KU Leuven, Kapucijnenvoer 7 block h, box 7001, 3000 Leuven, Belgium; 2 Division General Internal Medicine, Department of Medicine, University Hospitals of Geneva, Geneva, Switzerland; 3 Department of Health Research Methods, Evidence, and Impact, McMaster University, Hamilton, ON Canada

**Keywords:** Decision aids, Patient decision aids, Digital, User testing, Think aloud, Interview, Qualitative research, General practitioner, Primary care, Patient-centered communication, Patient participation, Shared decision making, Evidence-based medicine

## Abstract

**Background:**

Encounter decision aids (EDAs) are tools that can support shared decision making (SDM), up to the clinical encounter. However, adoption of these tools has been limited, as they are hard to produce, to keep up-to-date, and are not available for many decisions. The MAGIC Evidence Ecosystem Foundation has created a new generation of decision aids that are generically produced along digitally structured guidelines and evidence summaries, in an electronic authoring and publication platform (MAGICapp). We explored general practitioners’ (GPs) and patients' experiences with five selected decision aids linked to BMJ Rapid Recommendations in primary care.

**Methods:**

We applied a qualitative user testing design to evaluate user experiences for both GPs and patients. We translated five EDAs relevant to primary care, and observed the clinical encounters of 11 GPs when they used the EDA with their patients. We conducted a semi-structured interview with each patient after the consultation and a think-aloud interview with each GPs after multiple consultations. We used the Qualitative Analysis Guide (QUAGOL) for data analysis.

**Results:**

Direct observations and user testing analysis of 31 clinical encounters showed an overall positive experience. The EDAs created better involvement in decision making and resulted in meaningful insights for patients and clinicians. The design and its interactive, multilayered structure made the tool enjoyable and well-organized. Difficult terminology, scales and numbers hindered understanding of certain information, which was sometimes perceived as too specialized or even intimidating. GPs thought the EDA was not suitable for every patient. They perceived a learning curve was required and the need for time investment was a concern. The EDAs were considered trustworthy as they were provided by a credible source.

**Conclusions:**

This study showed that EDAs can be useful tools in primary care by supporting actual shared decision making and enhancing patient involvement. The graphical approach and clear representation help patients better understand their options. To overcome barriers such as health literacy and GPs attitudes, effort is still needed to make the EDAs as accessible, intuitive and inclusive as possible through use of plain language, uniform design, rapid access and training.

**Trial registration:**

The study protocol was approved by the The Research Ethics Committee UZ/KU Leuven (Belgium) on 31–10-2019 with reference number MP011977.

**Supplementary Information:**

The online version contains supplementary material available at 10.1186/s12911-023-02186-4.

## Background

In the past decades, the practice of medicine has witnessed a paradigmatic evolution from paternalism to increased patient empowerment and partnership [1–3], in response to the ethical and legal concerns of patients regarding their rights to being informed on their health and treatment [4]. This evolution culminates in shared decision making (SDM), which moves beyond patient information and consent as it requires a genuine involvement in actual decision-making [1, 2, 5]. Applying SDM and patient-centered communication in clinical encounters has shown to improve health-related patient outcomes [6]. Despite the benefits of SDM, its application in daily clinical practice remains a challenge [7, 8].

Patient decision aids (PDA) are tools developed to facilitate participation of patients making a decision by providing evidence-based information on benefits and harms of different options and by helping patients to clarify their values and preferences [9]. They exist in many formats that range from paper-based aids to computer-based, interactive decision tools [9, 10]. The PDAs can be used in face-to-face encounters (these tools are sometimes called encounter decision aids [11], the clinical encounter or through telephone or other communication media [12]. PDAs improve the quality of decision making by improving patient knowledge and reducing decisional conflict [13]. They increase patient participation and involvement and patients also have a more accurate risk perception [13, 14]. When used during the consultation, PDAs tend to increase assessment and satisfaction with the decision-making process [15].

Despite these findings, adoption of decision aids has been limited, as they are hard and onerous to produce, and typically not available for a broad range of decisions. They should respond to internationally adopted standards, and should convey the most correct and up-to-date knowledge [16]. However, a 2013 assessment found, even though mostly based on systematic reviews or guidelines, many of the PDAs were based on sources of questionable quality, had no expiry date or had no updating policy [17, 18].

**Fig. 1:**
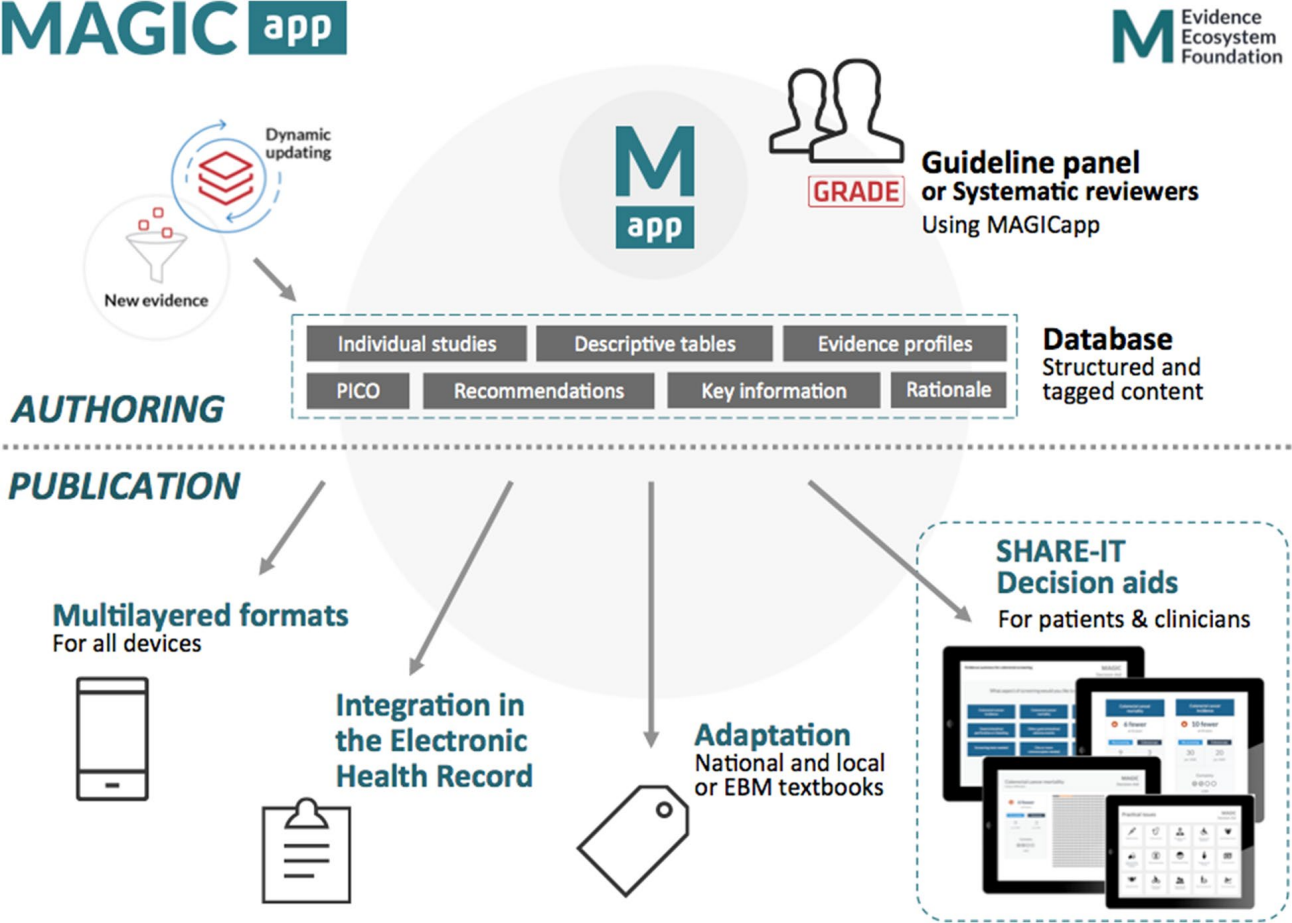
Generation of SHARE-IT encounter decision aids through the MAGICapp authoring and publication platform. Figure from Heen et al. [19] licensed under CC BY 4.0

In response to some of these challenges, the MAGIC Evidence Ecosystem Foundation has created a new generation of encounter decision aids (EDAs) that are generically produced along digitally structured guidelines and evidence summaries, in an electronic authoring and publication platform (MAGICapp) (Fig. 1). This allows a joint production and automatic updating, whenever the evidence is updated. The framework and initial user testing of these SHARE-IT decision aids (for “*Sharing Evidence to Inform Treatment decisions*”) has been described elsewhere in detail [18, 19]. But briefly, MAGICapp translated GRADE summary of finding tables into multilayered EDAs, allowing patients and clinicians to navigate across benefits and harms of interventions, as well as their practical issues, during the clinical encounter [20]. A recent evaluation and refinement of SHARE-IT decision aids showed promising results in real life utilization, thus providing a proof of concept of this approach linked to clinical practice guidelines [19].

Further, iterative evaluation and refinement of these EDAs in different contexts, such as a primary care setting, remain however indispensable in answering to barriers and facilitators of further usage [18, 21]. Despite the existing evidence concerning decision aids and their impact on healthcare quality, implementation in daily clinical routine remains difficult [22–24]. As qualitative and substantiated these tools might be, their usage remains dependent on the individual physician and his or her patient [25].

In this study we aimed to evaluate the experiences of both patients and their GPs when using SHARE-IT encounter decision aids in a primary care setting. We opted to use and test five EDAs all linked to the *BMJ Rapid Recommendations (i.e. Rapid Recs)* in primary care. The Rapid Recs are led by the MAGIC Evidence Ecosystem Foundation in collaboration with the British Medical Journal (BMJ) in response to practice changing evidence [26]. Our secondary objective was to identify barriers and facilitators to using the decision aids and aim to propose recommendations on future development or refinement of these tools.

## Methods

### Study design

We applied a qualitative user testing design to evaluate user experiences of general practitioners (GPs) and patients using the digital SHARE-IT encounter decision aids in real clinical encounters. We conducted a semi-structured interview with the patients after each encounter, as well as one think aloud interview with each GP after some encounters. The study took place in a primary care setting in Flanders, Belgium. Data was collected by 10 GP-trainees as part of their three-year postgraduate program, from October 2019 until May 2021. We performed this research simultaneously with the user testing of the infographics of the Rapid Recommendations, which were the source of the EDAs tested here (Van Bostraeten P, Aertgeerts B, Bekkering T, Delvaux N, Dijckmans C, Ostyn E, et al: Infographic summaries for clinical practice guidelines: results from user testing of the bmj rapid recommendations in primary care, unpublished).

### Intervention

From all published BMJ Rapid Recommendations (www.bmj.com/rapid-recommendations), we selected five online EDAs based on their relevance for general practice: thyroid hormones treatment for subclinical hypothyroidism [27], prostate cancer screening [28], antibiotics for uncomplicated skin abscesses [29], corticosteroids for treatment of sore throat [30], and arthroscopic surgery for degenerative knee arthritis and meniscal tears [31]. We translated the EDAs from English to Dutch using a forward–backward translation method. The forward translation was performed by five GP-trainees whereas the backward translation was done by an independent professional translator. We then reimplemented these translations into the MAGICapp platform, to make the Dutch version available with all the interactive features. We also made them available through *ebpracticenet* which is a national electronic point-of-care information service where health care professionals get free access to an up-to-date database of local and international guidelines and other evidence-based sources [32]. The main features of the EDAs can be found in Table 1. The links to the EDAs can be found in Additional file 1.

**Table 1:** Main concepts and features of the EDAs. Adapted from Heen et al. (2021) [19]

**Fig. 2:**
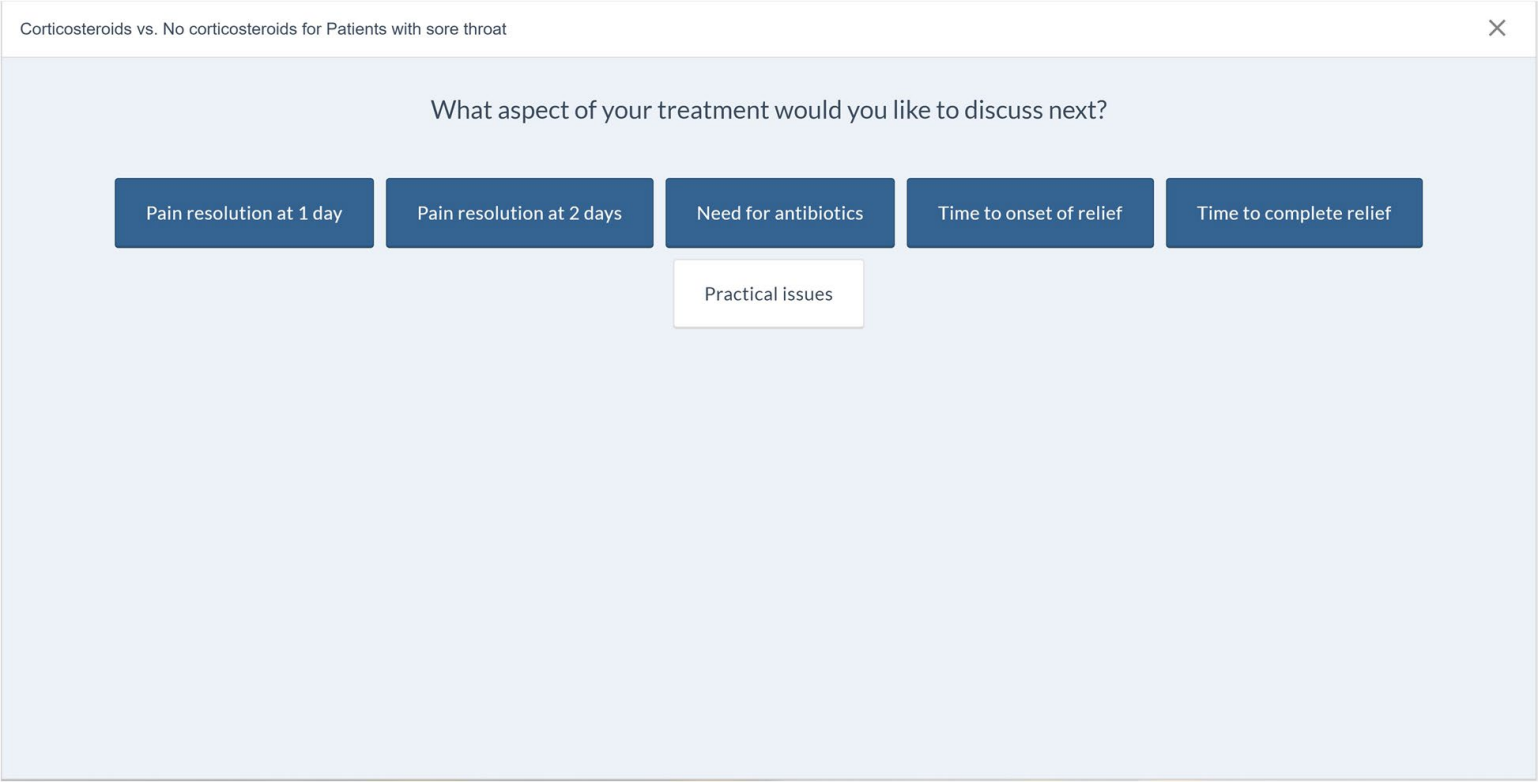
First layer with patient-important outcomes and practical issues. Figure from Heen et al. [19] licensed under CC BY 4.0

**Fig. 3:**
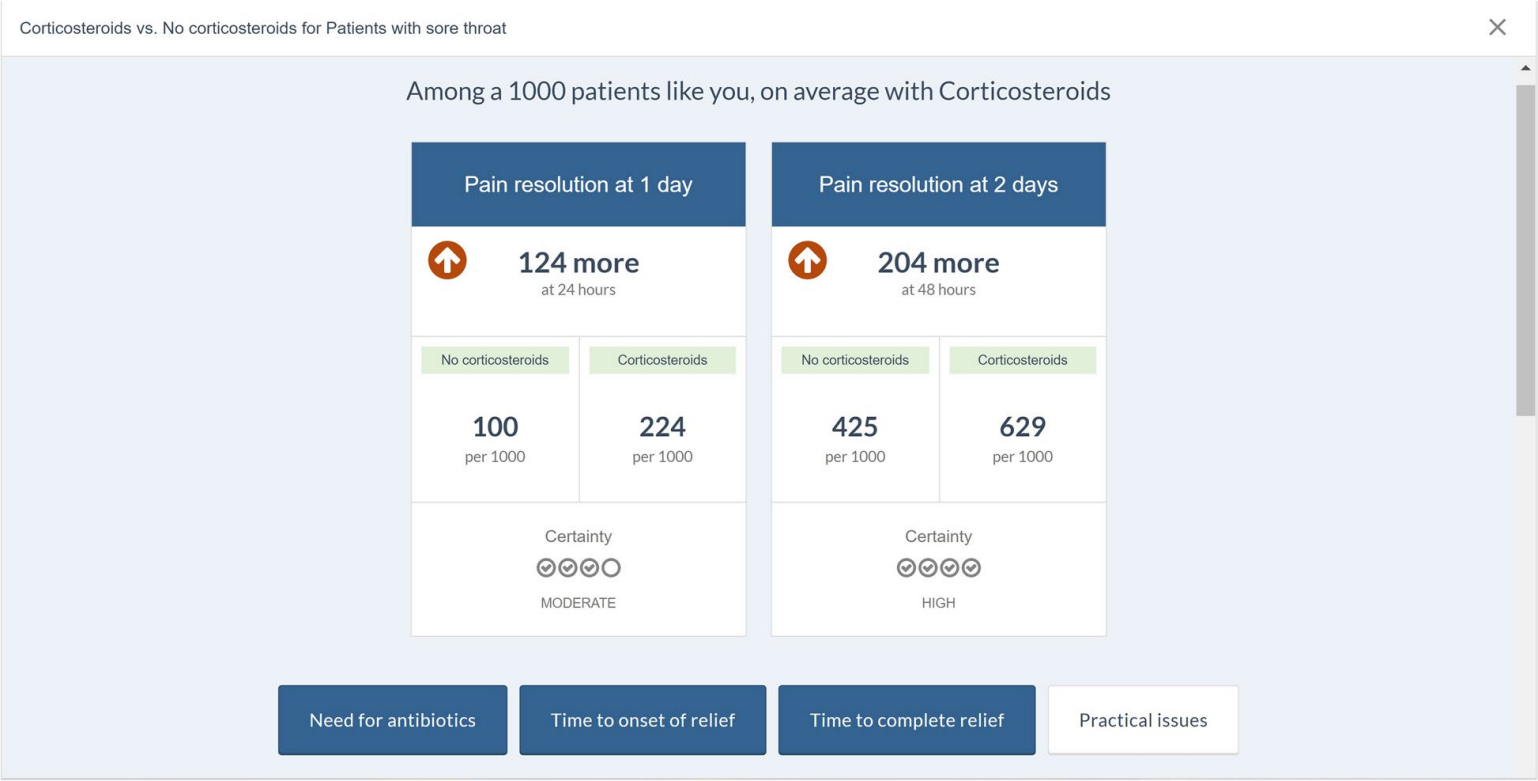
Second layer comparing outcome cards. Figure from Heen et al. [19] licensed under CC BY 4.0

**Fig. 4:**
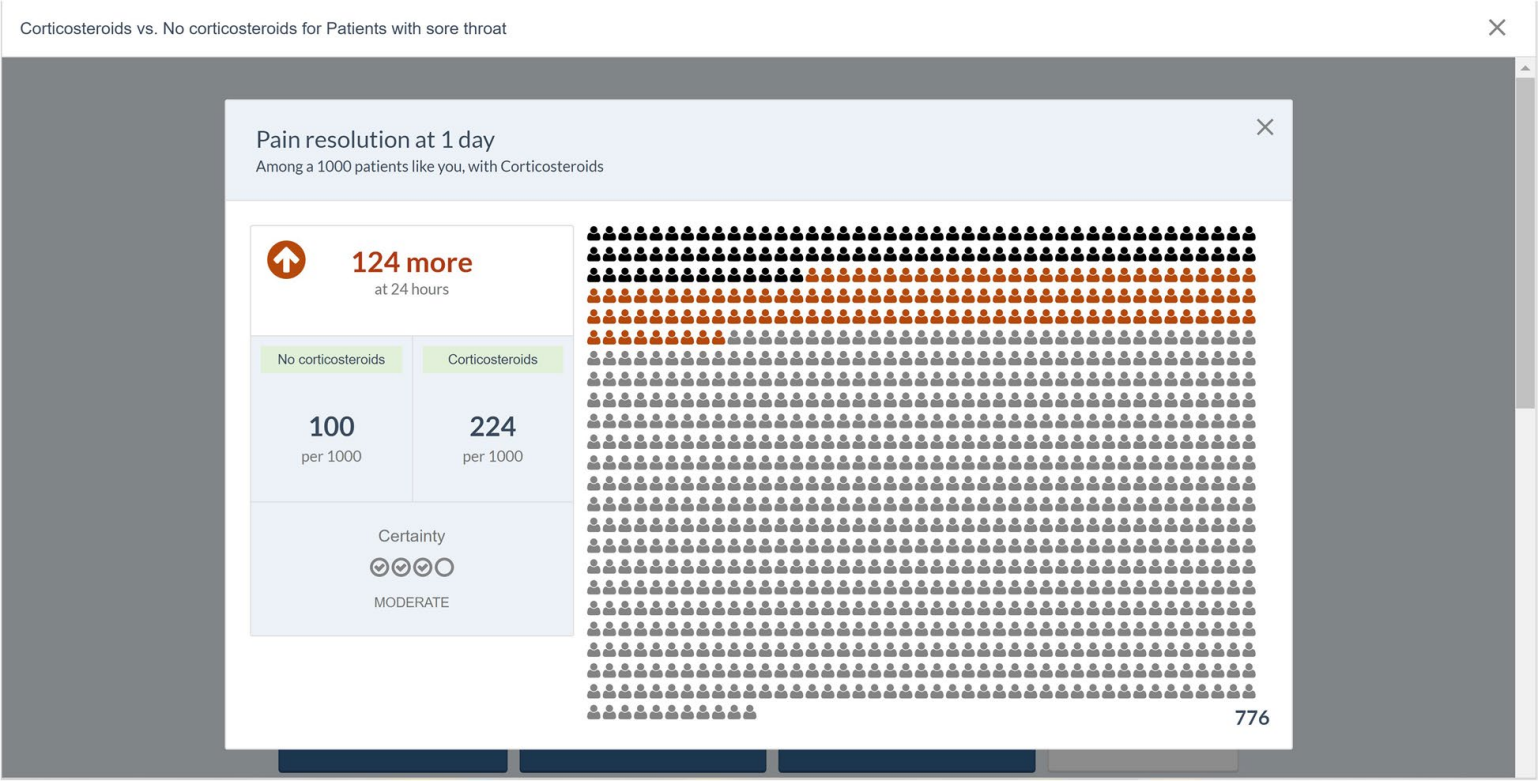
Third layer with pictographs showing the absolute risks. Figure from Heen et al. [19] licensed under CC BY 4.0

**Fig. 5:**
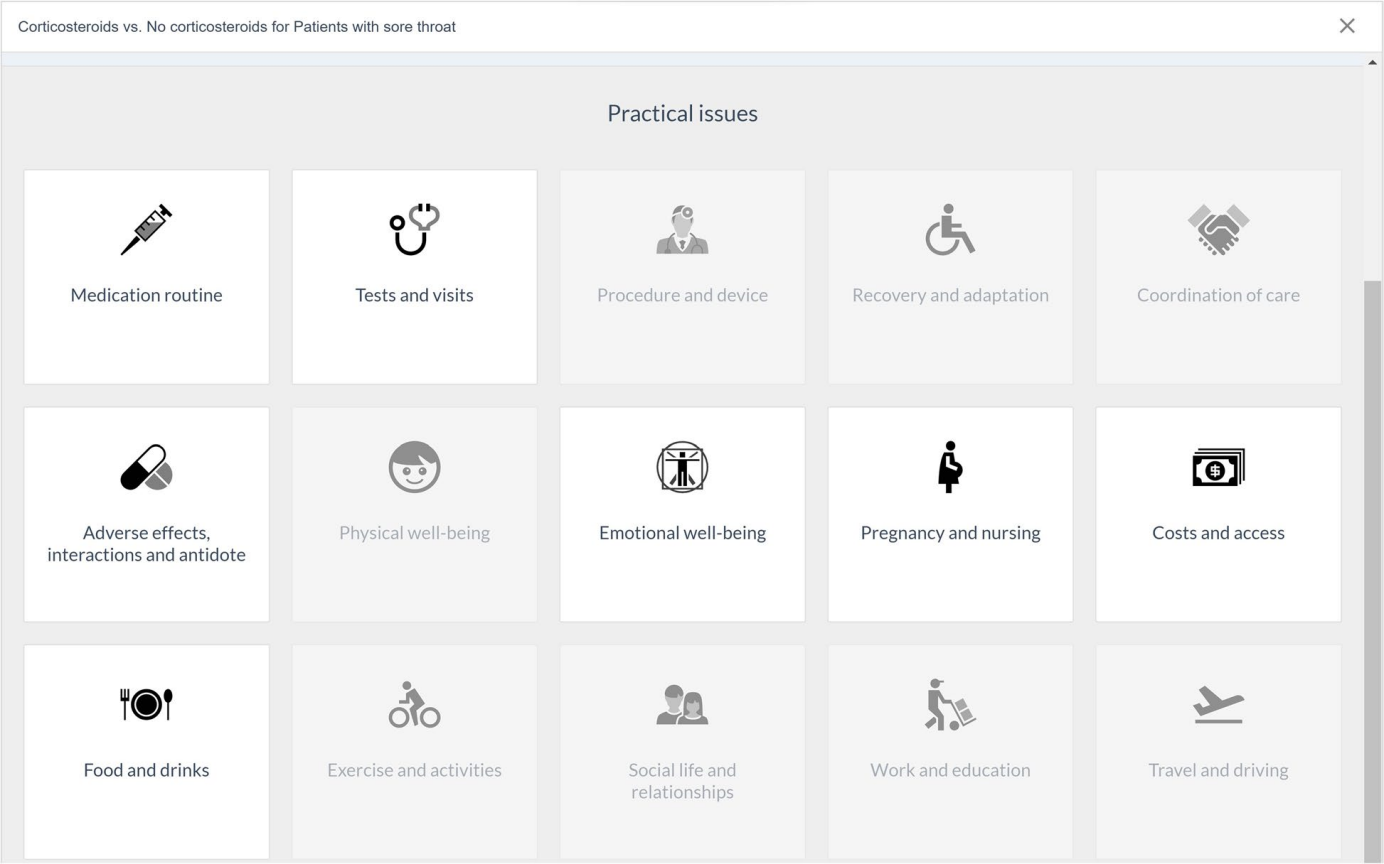
Third layer with practical issues related to the intervention. Figure from Heen et al. [19] licensed under CC BY 4.0

- Electronic generic framework for decision aids integrated in an authoring and publication platform for guidelines and evidence summaries (MAGICapp)
- Decision aids are semi-automatically produced and updated based on content in MAGICapp with adaptation possibilities (e.g. wording and number of outcomes, language)
- Multi-layered presentation format: ○ First layer displays the list of patient-important outcomes and practical issues. (Fig. 2) ○ Second layer displays interactive outcome cards with evidence estimates, certainty, and patient-important practical issues across 15 generic categories. Possibility to interactively compare two or more outcomes in parallel. (Fig. 3) ○ Third layer displays a corresponding set of pictographs showing the absolute risk with each option (Fig. 4) and practical issues related to the treatment option (Fig. 5)
- Educational module developed http://magicproject.org/161128/ and integrated in MAGICapp. This module was not used in our study, yet could be accessed by the GPs on their own
- Print functionality of decision aids create pdf files that can be printed or used for notetaking and/or to bring home
- Prototype for comparisons between multiple options are developed and implemented in a BMJ Rapid Recommendation
- Offline feature so decision aids can be used without use of Internet
- Widgets from MAGICapp to grab and show a given decision aid on any other online platform. Example: Rapid Recommendation on Prostate cancer screening (https://www.bmj.com/content/362/bmj.k3581 to BMJ infographic) which links to MAGICapp content, including widgets to decision aids for various profiles of patients

Prior to the test phase the GPs were familiarized with the *BMJ Rapid Recommendations* and associated EDAs. They were instructed through a 1.5 h online training on the study design and on how to use both the *RapidRecs* and the EDAs by the research team.

The real-life user testing happened during and after clinical encounters between patients and their GPs, observed by GP-trainees. When a decision related to one of the selected EDAs had to be made, the GP accessed the corresponding EDA through *ebpracticenet* and used it as instructed. The EDAs were used during the encounter on the computer screen of the GP. Patients did not have to access the EDA before the consultation.

### Participants, recruitment and setting

We recruited GP-trainers through the GP-trainees contributing to this project, working in the same practices, by phone or by invitation letter. We excluded GPs who did not speak Dutch. We aimed to recruit at least 10 GPs with a heterogeneity in gender, age and geographic distribution. We recruited patients during real patients encounters or by phone when an appointment was planned concerning one of the relevant topics. Every patient was informed through an information letter and by the recruiter orally. We obtained informed consent for every participant. Patients could either choose to participate right away or make a new appointment if they wanted to think about participation.

### Data collection

Following the clinical encounter, we conducted one-on-one, semi-structured interviews with the patients (see Additional file 2). We also interviewed the GPs in a one-on-one think-aloud session, asking them open questions after having used the EDAs during at least three clinical encounters [33, 34]. All interviews were recorded by audio or video and transcribed verbatim. Consultations were either attended by one of the researchers or were recorded and viewed afterwards to observe usage and to be able to provide feedback during the interviews. All data was collected between October 2020 and January 2021.

### Data processing

Audio-recorded interviews were not distributed and kept secure by the respective interviewer. Verbatim transcriptions were made anonymously and references to the identity of the interviewed GP or patient were avoided. Certain characteristics (age, gender, type of practice, etc.) were mentioned on the transcriptions as they were believed to contribute to the quality of the subsequent analysis. Transcriptions were uploaded in a shared project on the software program Atlas.ti [35] and were not distributed elsewhere.

### Data analysis

**Fig. 6:**
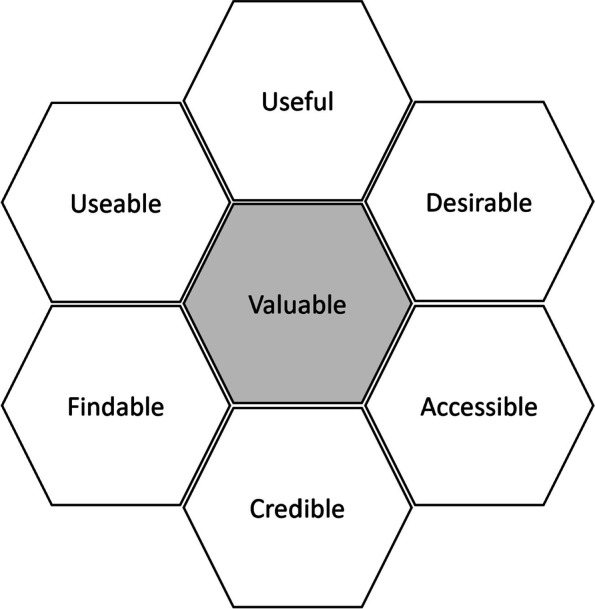
Morville’s facets of user experience – honeycomb [39]

Analysis was performed using the Qualitative Analysis Guide of Leuven [36]. Transcripts were uploaded to a shared research project in Atlas.ti Cloud [35] and the research team was divided into two groups, one for GP interviews and one for patient interviews. Analysis was conducted by the researchers working in pairs. The first researcher coded a transcript and the second re-coded, checking for any discrepancies. Discrepancies were discussed until consensus was achieved by the whole research group. A second analysis was performed by 3 researchers (PVB, LM, JJ) among who two that were not involved in the initial data collection, to gather different and independent insights. Coding was performed using a three layered structure. First, we coded for subjective thoughts by labelling sentiment (i.e. positive, minor frustrations to “show stoppers” and suggestions). Second, all notes were classified into overall concepts which were created inductively through team collaboration. The third layer involved deduction to the six different categories of the Morville’s honeycomb model: usability, usefulness, desirability, findability, accessibility and credibility (Fig. 6, Table 2) [37, 38].

**Table 2:** Morville’s facets of user experience – definitions [38, 39]

Facet	Explanation
Usability	Refers to how simple and easy to use the product is. The product should be designed in a way that is familiar and easy to understand. The learning curve a user must go through should be as short and painless as possible
Usefulness	Refers to how much the product fills or answers an information need. If the product is not useful or fulfilling the user's wants or needs, then there is no real purpose for the product itself
Desirability	Refers to the visual aesthetics of the product, which needs to be attractive and easy to translate. Design should be minimal and to the point
Findability	Refers to how easy to navigate the product is. If the user has a problem they should be able to quickly find a solution within the product, and the navigational structure should also be set up in a way that makes sense
Accessibility	Refers to how accessible and adapted the tool is, even to users with special needs, so that they can have the same user experience as others
Credibility	Refers to how trustworthy the product is. Note that this may refer to the product itself, as well as to content that informs it (which is not necessarily an attribute of the design)

### Ethics approval

The study was reviewed and approved (mp011977) by the research ethics committee UZ/KU Leuven. There was no financial reward for participants in the study.

## Results

### Overview of user experiences

We performed 31 interviews with patients and 11 think-aloud sessions with GPs. Of the patients, 19 were men and 12 were women with age ranging between 20 and 88 years. Of the GPs, 6 were men and 5 were women with age ranging between 29 and 70 years. 1 GP worked solo, 5 worked in duo and 5 in a group practice. 4 GPs worked in a city environment while 7 worked in a more rural environment.

Patients and GPs reported most on the dimensions of usability, usefulness, and desirability. Most of the reports were expressions of positive feedback for both groups. Table 3 provides a summary of the results.

**Table 3:** Summary of results

Honeycomb	Finding
Usability	Visual representation supports understanding of the content
	A learning curve exists before fluent use of the EDAs
	A new consultation format arises that needs to be adopted
	Difficult terminology reduces understandability for both patients and GPs
	Both patients and GPs worry about the time required to use the EDA
	The EDAs do not always apply to the personal situation of the patient
	Patient’s characteristics determine whether EDAs are used or not
	GPs may feel pushed to discuss difficult topics they prefer to avoid
Usefulness	The EDAs provide necessary information and insights to support the shared decision making process
	Patients can identify due to description of complaints they experience
	There is a thin line between providing the right information and providing too much information that is too specialist
	Patients gained more trust in their GP due to the use of an EDA
	EDAs are perceived less useful when they differ from local guidelines
Desirability	EDAs are enjoyable tools to work with
	By having an interactive, multilayered nature, the EDAs remain clear
	Uniformity in lay out between EDAs is desired
	The location of the topic indicates its importance (e.g. the first topic seems more important than the last one)
Findability	Some patients would like to use the EDA at home, before or after the consultation
	Difficulty finding the tool might prevent from future use
Accessibility	Terminology in a foreign language makes the tool more difficult to use for patients
	An interactive, multilayered structure helps reducing an overwhelming amount of information
Credibility	Patients trust the EDA when it is used by their GP
	GPs trust the EDA due to a trustworthy source
	The EDAs are less used when they are not in line with the GP’s own views or experiences

### Usability

#### Patients

In general, patients experienced the EDAs as very usable in the consultation as it was clear, understandable and generally simple to use. Some patients needed to get used to the new form of consultation, with shared decision making mediated by a decision aid, and some were more reluctant due to being accustomed to the old consultation model. Some patients mentioned that the use of the EDAs could be more fluent if and when the GP was more experienced with it.

“Personally, I found the information to be clear and very well brought.”

“Probably, yes. But as I said, I still need to adapt a bit.”

Certain unfamiliar terms (e.g. ‘meniscus’, ‘arthroscopy’, ‘adverse events’) were difficult to understand, as were many numbers and risks. A few stated that it was difficult to understand how the many numbers displayed actually applied to their own personal situation. For both terms and numbers, patients found it important that there was assistance by their GP and they stated that the EDA could not replace the consultation. However, some fear their GP might lose precious time using the tool.

“It is very difficult to interpret the importance of a ‘0,1’, you know. (…) is it much, is it little? If you’re not into that terminology, you just don’t know.”

“ (…) a few times I thought: ‘okay… meniscus, arthroscopy, … this is this and that is that’. Doctor x eventually explains it very well and very calmly.”

The visual pictographs, displayed in the third layer of the EDA, were considered very helpful. Some patients would have liked more explanation about the disease as an introduction or would have liked the ability to prepare themselves before the consultation.

“Well, here you see only numbers, but if you use bars, you see it better, you know. How much … you know. That’s what I mean.”

#### General practitioners

Most GPs got used to the tool quickly and stated the tool as being easy to use. Nevertheless, many GPs experienced some difficulties. The interpretation of certain terminology (e.g. ‘thyroid-related symptoms’) or certain numbers was difficult, because of lack of accompanying explanation. This information could most of the time be found in the infographic displayed in the associated guideline, although GPs preferred a more direct link to the infographic as it was too cumbersome to find it fast in the consultation. Moreover, GPs found it difficult to give meaning to the certainties of each outcome (i.e. GRADE certainty in the estimated of effect [40]).

“Pff, thyroid-related symptoms… Maybe put in a short explanation of which symptoms because it’s not there.”

GPs agreed that their support was needed for the patients going through the decision aid. They found that some terminology was too difficult to understand for patients (e.g. ‘cardiovascular events’). They stated that this should be avoided and that a patient should not be overestimated when it comes to medical knowledge. Some even took on a more paternalistic role to guide the patient through the EDAs on the presumption they’re better positioned to evaluate their patient’s ideas, concerns and expectations.

“ Yes, some terminology is quite vague, isn’t it? Cardiovascular events, we know what it is but patients do not understand a bit of it, you know.”

“ But I think the patients do not want to decide themselves. Even they expect some decisiveness and directivity from the physician, I think.”

Some GPs thought the integration of the EDAs in their consultation was time-consuming. They experienced that the use of the decision aids required a certain amount of training, and skill, subjective to an individual learning curve. Some stated this could be an argument to not use the EDAs. When patients consulted with more than one issue for example, some GPs mentioned they would less likely use the EDA.

“When you feel it will take you more time than it will generate, I think you will be tempted to not use it anymore.”

The patient's characteristics (e.g. control of the Dutch language, education, individual habits, …) played a role in the GPs decision, whether or not to use the EDAs. For that matter, some GPs expressed the preference of EDAs in the mother language of their patient as they felt it would improve the quality of the consultation.

“I assess the intellectual level of the patient. Whether he’s susceptible, sensitive to it, whether he can agree with it. Not all people can appreciate this, some will most likely say: ‘I come to you for this’.”

Some GPs found the EDAs to be too theoretical, losing sight of more practical matters. They found it difficult to apply statistics to the individual and were sometimes withhold when labelled as ‘weak recommendation’. To be more practical, some GPs suggested to define the population more strictly (e.g. ‘degenerative knee disease’).

“Statistics are really good, but it doesn't say anything about one individual.”

Finally, some GPs felt uncomfortable discussing more serious risks such as mortality and serious adverse events with their patients which they wouldn’t do systematically without the EDAs.

### Usefulness

#### Patients

Patients enjoyed receiving more information than they were used to in other consultations. It resulted in better insight into the illness and various treatment options, which supported them to make better decisions. Patients felt more involved in the consultation as the EDA offered them an individual approach based on their own concerns and interests. They could identify more as they could recognize certain complaints mentioned in the EDA. Patients were more reassured after the consultation and had gained trust in their GP. Some patients wished the EDA was used more in the consultation and others wanted to be able to use it at home.

“He explored my own personal problem, what I was most concerned about.”

Opinions were more divided regarding the quantity of the information, as some were fine with the amount, some wanted more and some found the amount to be overwhelming. It seemed to be important that the information answers the needs of the patient.

“I find this excellent, very enriching to me. Now I have even more information.”

“At first, it's a bit overwhelming.”

#### General practitioners

GPs found the EDAs to be useful and helpful. They supported them in their consultation by conveying the existing evidence to reach an informed and shared decision that met the ideas, concerns and expectations of their patients. They were often found to create a more personal approach. Besides the consultation, it was said to even lead to new insights in primary care and was a way to keep up to date.

“The majority of subjects or questions in the mind of the patients are addressed.”

“Comfortable to have the numbers, when there was a question. If I can give him the right information, that will help and support a well-founded decision that we both support.”

About the content of the EDAs was some discussion. Some GPs felt the EDAs included information that was too specialized and that a lot of less relevant issues were discussed. When patients asked for more information about these specialized topics, GPs felt awkward for they could not answer and the information was not found on the EDAs.

*“When he would ask me as a doctor – which he actually practically did – what might then be the cause of death? Well, I didn’t know either. I couldn’t find in it (the tool) which answer to give.”*

GPs stated that some EDAs lost their usefulness when they differed from locally established guidelines, e.g. use of antibiotics, or when they were not in line with their own views or past experiences of the topic.

“… I would sometimes prescribe ‘Amoxiclav’ or ‘Floxapen’ and it was not an option. So I wonder why it’s not an option?”

Most GPs found the tool to be useful for future use. One GP wanted that the use of EDAs would become integrated in the medical curriculum.

“I think such patient decision aids are what we have to strive for more and more as a doctor.”

### Desirability

#### Patients

In general, patients were pleased with the design of the EDA as it was very clear and organized. Most patients enjoyed the EDAs and would like them to be used in future appointments. Patients liked the visual representation and pictographs of numbers and risks, as it helped them better understand their meaning. Patients found it pleasant and important that the tool was interactive as this preserved overview.

“Personally, I found the information very clear and well brought.”

“The presentation with these little men is brilliant!”

#### General practitioners

The interactive nature of the tool was experienced as very appealing as it afforded the ability to choose topics individually. The visual representation led to a more involved patient that seemed to understand the information better. The EDA with its multiple subcategories was clearly organized for most. Some would have liked to see more figures and more contrasting colors and wanted more uniformity in design between the different EDAs so they needed less adopting when using a new one. Finally, many GPs advised to sort topics by relevance and by impact (e.g. mortality not as first).

“I think it’s clear, uhm, in terms of graphic lay-out.”

About ‘death’ as first topic: “It can certainly be mentioned, but I might, … more at the end, move it a little back.”

### Findability

#### Patients

Some patients would have preferred to be able to reread the information as shown in the EDA after the appointment with their GP.

“That would be easy if you could review it at home.”

#### General practitioners

Some found the EDAs to be easily available online, while others indicated they had trouble finding the EDAs on their own.

“Online, nearly on the spot, information ready to use!”

“They are just difficult to find.”

### Accessibility

#### Patients

The tool seemed to have little difficulty in accessibility, as it was clear in regard to form and presentation. Due to some errors in the translation process, not all terminology was translated to Dutch in the final version, especially in the deeper layers of the EDAs. When confronted with English terminology, some patients became confused and mentioned that the use of terms in a foreign language should be avoided. Some patients lost overview when too much information was opened and wished for it to be opened and closed in a more step-by-step fashion. Most patients expect their GP to assess which information is relevant for them.

“An explanation can be overwhelming, but when presented in a clear fashion on a computer, I find it more comforting.”

#### General practitioners

Most GPs found the EDAs easy to use and there were no issues with readability. GPs stated that the ease of use was very important to them if they wanted to use the EDAs more. One recommendation was that the tool should be easily and rapidly accessible. One GP mentioned a difficulty of use due to the inability of turning her computer screen to the patient.

Some GPs had the impression that the information could be overwhelming for specific patients and that a clear overview is very important. The GPs stressed the importance of not straining their patients with too many different categories, or figures.

*“I think you should be careful not to overload the patient with too many figures. I hope I'm wrong, but I think we sometimes overestimate the patient. Certainly on a medical, anatomical (refers to anatomy) and numerical level.”*

### Credibility

#### Patients

Patients trusted the tool as it was provided by their own GP. They gained more trust in the decision they took as the presence of objective data created confidence. Some patients also even indicated their trust in their own GP had increased due to the use of the EDAs and found their GP to be modern and up to date.

“This has given me more confidence in the decision we took together.”

“Certainly good, my trust (in my physician) increased a little.”

#### General practitioners

The GPs found the EDAs to be trustworthy as they were provided by a respectable source. In this case these were the university, the BMJ and ebpracticenet. Some GPs lost their trust in certain EDAs when they were not in line with their own experiences.

“Of course, it’s important that we know it’s developed in the context of a master’s thesis, or by the university.”

## Discussion

### Main findings

The overall experience of using an EDA in a real-life clinical encounter was positive for both patients and GPs. They felt it supported the shared decision making process as it enhanced involvement in the consultation, as well as a more efficient and understandable transmission of information on each treatment alternative. Patients appreciated the time spent on them and expressed more trust towards their GP.

The EDAs were found to be easy in use, though both patients and GPs felt that some learning and experience was needed. Time investment both in- and outside of the consultation was therefore a concern. Uniformity between EDAs was recommended to reduce the learning curve for each EDA.

Patient characteristics and GPs beliefs were the main barriers for using the EDAs. GPs noticed an importance of patient characteristics such as health literacy to be able to use the decision aids. For that matter, GPs mentioned that sometimes too technical terminology was used. Both patients and GPs recommended more simple language, preferably provided in the mother language of the patient. Regarding understandability, GPs preferred some more explanation about certain terminology with which they are not familiar. A direct link to the corresponding guideline was proposed. GPs did seem to trust most information due to it being brought by a trustworthy source, such as the university or the BMJ. The deviation from certain common local guidelines however, such as nationally established antibiotics regimens, were considered an important issue.

The design was experienced as very appealing. The interactive approach compensated the amount of information that was sometimes still overwhelming. The visual representation aided in more understanding. GPs would have preferred more serious outcomes (mortality, sepsis) to be listed rather near the end and not at first, as they feared it might shock their patients. The tool was easily accessible on a pc, though GPs recommended a more rapid access such as through the electronic health record.

### Strengths and limitations

We were able to gather data from a varied population of both GPs and patients. Our results are based on a good representation of patients that GPs see in their day-to-day practice and a broad range of experiences and feedback was obtained.

Our analysis was rigorous, as each interview was thoroughly analyzed by at least two researchers and afterwards discussed and reflected upon by the whole research team. This detailed approach ensured that none of the data got lost in the process. To make sure our data is complete and structured, we applied Morville's Honeycomb method (Table 2).

By analyzing experiences of both physicians and patients and checking them on observations from researchers who were not involved in the consultation, we feel we were able to synthesize an integral view on the user experience of the encounter decision aids, as a multiple perspective assessment appears the best approach in SDM [41, 42].

One limitation to our study is that it was conducted in the practices where the researchers were themselves working at the time. This may have prevented truly honest feedback, especially while reporting negative features. Another limitation is that some errors occurred during the translation of the EDAs, where certain terminology remained in English. This might have impacted the experience, though it also provided us with interesting results. Finally, we also faced some difficulties while conducting the research due to the COVID-19 pandemic. Due to this busy period, the time GPs could devote to preparation and execution could have been limited and the obligation of wearing a mouth mask might have influenced the interaction of the consultation.

### Comparison with other literature

A greater involvement of the patients and an increase in the ability and confidence on making their own decision was observed. This is in line with a recent Cochrane review comparing usual care to the use of patient decision aids in practice [13]. Patients are more knowledgeable, better informed and clearer about their values when evaluating the different options in regard to usual care. When exposed to patient decision aids, patients experience an increase in satisfaction and perceived involvement [43]. These decision aids help the physician to inform the patient about existing, reasonable choices and help to explain and generate accurate expectations of benefits and harms, which are two of the three main pillars of the ‘Three-Talk Model’ of shared decision making [44]. They are helpful tools yet can and should not completely cover the full shared decision making process [43, 45].

We must be careful when interpreting the patient’s evaluation of involvement. We felt patients did not always understand the full concept of shared decision making and answered often positively due to respect towards their GP. It is true that a respectful and encouraging relationship with the provider influences the sense of involvement in decision making [46]. Our observations however, are strongly suggestive that there was actually a greater involvement during the consultation.

Some terminology and interpretation of scales and grades was found to be difficult by both patients and GPs. Physicians and patients are not always experienced in evaluating the meaning of certain risks and different formats of presentation can create bias [47, 48]. Visual aids, graphs, or pictures and a more concrete presentation of numerical information show promising results in supporting patients to better understand benefits, harms and practical issues of treatment options [20, 48–50].

Patient characteristics, such as low health literacy and age, were perceived as barriers for using the EDAs. A patient's ability to engage in shared decision making is largely determined by their health literacy skills [51]. Previous research however indicates that shared decision making interventions may be more beneficial for groups with lower health literacy and/or socioeconomic status [52]. Moreover, scarce research shows promising results for decision aids in elderly as they improve their knowledge, risk perception and participation in the shared decision making process while decreasing decisional conflict [53]. By withholding these interventions from those who need it the most, an even greater gap in health care is created. Attention should hence be brought to adapting the decision aids in a manner that they are inclusive to everyone. One should use plain and adjusted language to support an optimal transfer of information [54–56].

Time management seemed to occupy both GPs and patients. In the before mentioned Cochrane review, a median increase of 2.6 min was seen on a median consultation of 21 min, yet 8 out of 10 studies showed no difference with usual care [13]. Preparation time is however not taken into account for the duration of the consultation and might contribute to a bigger time investment, at least initially. Difficult findability is another factor that might increase time consumption and integration in the electronic health record might be helpful. This solution has been proposed before as well and the electronic health record is believed to be a potentially powerful tool for promoting shared decision making [57]. Still, some GPs were insecure in being able to find the decision aids on themselves when not provided by us. A reason might be that the use of decision aids is not yet established as usual practice and GPs are not trained on how and where to access decision aids. Education and integration in undergraduate medical curricula can offer support regarding this issue [58]. Another study design, where decision aids are not provided by the researchers, would gain us more insight in findability related issues.

### Recommendations for future research and development

Further research could be done to reduce the learning time GPs are experiencing. EDAs should be made very intuitive and uniform in presentation. A clear, short teaching module could be developed. Integration of EDAs in the medical curriculum could support future generations.

Great investment should be put in making the EDAs accessible and understandable for everyone, despite their personal characteristics. Plain language should be the standard. Availability in the mother language would be a great asset. Explanation ‘if needed’ should be provided, for patients as well as for physicians.

Rapid access is found to be necessary. Integration in the electronic health record might be a viable option.

User testing is particularly powerful when it is used as part of an iterative or cyclical design process in which designs are tested, modified and retested [59]. This study should hence be seen as part of a continuous process with each iteration necessitating further user testing. Further user testing could be particularly useful in:

- General practitioners with different characteristics (age, rural/urban employment, solo or group practice, …)
- Patients with specific backgrounds (age, health literacy, language barriers, …)
- Different countries with different organizations of care

## Conclusions

We evaluated the experience of GPs and their patients using digital encounter decision aids available in the MAGICapp. The overall experience was positive for both patients and GPs. Although some adoption is still needed, the decision aids were able to shift the consultation towards more patient involvement and patient-centered care as it supported shared decision making. Effort is still needed to make the EDAs as accessible, intuitive and inclusive as possible through use of plain language, uniform design, rapid access and training.

## Supplementary Information

**Additional file 1.** Links to EDAs.

**Additional file 2.** Semi-structured interview questions.

## Data Availability

The datasets used and/or analysed during the current study are available from the corresponding author on reasonable request.

## Abbreviations

QUAGOL: Qualitative Analysis Guide of Leuven
GP: General Practitioner
EDA: Encounter Decision Aid
PDA: Patient Decision Aid
SDM: Shared Decision Making
EHR: Electronic Health Record

## References

[1] Charles C, Gafni A, Whelan T. Shared decision-making in the medical encounter: What does it mean? (or it takes at least two to tango). Soc Sci Med 1997 44(5):681–92. Available from: https://linkinghub.elsevier.com/retrieve/pii/S0277953696002213. 10.1016/s0277-9536(96)00221-3 9032835

[2] Pieterse AH, Finset A (2019). Shared decision making—Much studied, much still unknown. Patient Educ Couns.

[3] Szasz TS (1956). A contribution to the Philosophy of medicine. AMA Arch Intern Med.

[4] Clapp JT, Fleisher LA, Lane-Fall MB (2019). Decision aids are a solution, but to which problem?. Anesth Analg NLM (Medline).

[5] Barry MJ, Edgman-Levitan S. Shared decision making — the pinnacle of patient-centered care. N Engl J Med 2012 366(9):780–1. Available from: http://www.nejm.org/doi/10.1056/NEJMp1109283. Cited 2022 Feb 28. 10.1056/NEJMp1109283 22375967

[6] Street RL, Makoul G, Arora NK, Epstein RM (2009). How does communication heal? Pathways linking clinician-patient communication to health outcomes. Patient Educ Couns.

[7] Couët N, Desroches S, Robitaille H, Vaillancourt H, Leblanc A, Turcotte S (2015). Assessments of the extent to which health-care providers involve patients in decision making: A systematic review of studies using the OPTION instrument. Health Expect.

[8] Stiggelbout AM, van der Weijden T, de Wit MPT, Frosch D, Légaré F, Montori VM, et al. Shared decision making: really putting patients at the centre of healthcare. BMJ 2012 344(7842). Available from: https://pubmed.ncbi.nlm.nih.gov/22286508/. Cited 2022 Feb 28. 10.1136/bmj.e256 22286508

[9] O’Connor A. Using patient decision aids to promote evidence-based decision making. BMJ Evid Based Med 2001 6(4):100–2. Available from: https://ebm.bmj.com/content/6/4/100. Cited 2022 Feb 28.

[10] Patient Decision Aids - Ottawa Hospital Research Institute. Available from: https://decisionaid.ohri.ca/. Cited 2022 Feb 28.

[11] Wyatt KD, Branda ME, Anderson RT, Pencille LJ, Montori VM, Hess EP, et al. Peering into the black box: A meta-analysis of how clinicians use decision aids during clinical encounters. Implement Sci 2014 9(1):1–10. Available from: https://implementationscience.biomedcentral.com/articles/10.1186/1748-5908-9-26 . Cited 2022 Sep 28. 10.1186/1748-5908-9-26 PMC3936841 24559190

[12] Elwyn G, Frosch D, Volandes AE, Edwards A, Montori VM. Investing in deliberation: a definition and classification of decision support interventions for people facing difficult health decisions. Med Decis Making 2010 30(6):701–11. Available from: https://pubmed.ncbi.nlm.nih.gov/21088131/. Cited 2022 Feb 28. 10.1177/0272989X10386231 21088131

[13] Stacey D, Légaré F, Lewis K, Barry MJ, Bennett CL, Eden KB (2017). Decision aids for people facing health treatment or screening decisions. Cochrane Database Syst Rev.

[14] Coronado-Vázquez V, Canet-Fajas C, Delgado-Marroquín MT, Magallón-Botaya R, Romero-Martín M, Gómez-Salgado J. Interventions to facilitate shared decision-making using decision aids with patients in primary health care: a systematic review. Medicine 2020 99(32):e21389. Available from: https://pubmed.ncbi.nlm.nih.gov/32769870/. Cited 2022 Feb 3. 10.1097/MD.0000000000021389 PMC7593011 32769870

[15] Scalia P, Durand MA, Berkowitz JL, Ramesh NP, Faber MJ, Kremer JAM, et al. The impact and utility of encounter patient decision aids: Systematic review, meta-analysis and narrative synthesis. Patient Educ Couns 2019 102(5):817–41. Available from: https://pubmed.ncbi.nlm.nih.gov/30612829/. Cited 2022 Feb 28. 10.1016/j.pec.2018.12.020 30612829

[16] International Patient Decision Aids Standards (IPDAS) Collaboration. Available from: http://www.ipdas.ohri.ca/. Cited 2022 Feb 28.

[17] Montori VM, Leblanc A, Buchholz A, Stilwell DL, Tsapas A. Basing information on comprehensive, critically appraised, and up-to-date syntheses of the scientific evidence: a quality dimension of the international patient decision aid standards. 2012; Available from: http://www.biomedcentral.com/1472-6947/13/S2/S5. Cited 2022 Feb 1. 10.1186/1472-6947-13-S2-S5 PMC4044946 24625191

[18] Agoritsas T, Heen AF, Brandt L, Alonso-Coello P, Kristiansen A, Akl EA, et al. Decision aids that really promote shared decision making: the pace quickens. BMJ 2015;350. Available from: https://www.bmj.com/content/350/bmj.g7624. Cited 2022 Feb 1. 10.1136/bmj.g7624 PMC4707568 25670178

[19] Heen AF, Vandvik PO, Brandt L, Achille F, Guyatt GH, Akl EA (2021). Decision aids linked to evidence summaries and clinical practice guidelines: results from user-testing in clinical encounters. BMC Med Inform Decis Mak.

[20] Heen AF, Vandvik PO, Brandt L, Montori VM, Lytvyn L, Guyatt G (2021). A framework for practical issues was developed to inform shared decision-making tools and clinical guidelines. J Clin Epidemiol.

[21] Hill L, Mueller MR, Roussos S, Hovell M, Fontanesi J, Hill J (2009). Opportunities for the use of decision aids in primary care. Fam Med.

[22] Joseph-Williams N, Abhyankar P, Boland L, Bravo P, Brenner AT, Brodney S, et al. What works in implementing patient decision aids in routine clinical settings? A rapid realist review and update from the international patient decision aid standards collaboration. Med Decis Making. 2020;41(7):907–37. 10.1177/0272989X20978208 PMC8474331 33319621

[23] Stacey D, Suwalska V, Boland L, Lewis KB, Presseau J, Thomson R (2019). Are patient decision aids used in clinical practice after rigorous evaluation? A survey of trial authors. Med Decis Making.

[24] Elwyn G, Légaré F, van der Weijden T, Edwards A, May C. Arduous implementation: Does the Normalisation Process Model explain why it’s so difficult to embed decision support technologies for patients in routine clinical practice. Implementation Science. 2008;3:57. Available from: http://www.implementationscience.com/content/3/1/57Cited 2022 Feb 1. 10.1186/1748-5908-3-57 PMC2631595 19117509

[25] Scalia P, Durand MA, Elwyn G. Shared decision-making interventions: An overview and a meta-analysis of their impact on vaccine uptake. J Intern Med 2021; Available from: https://pubmed.ncbi.nlm.nih.gov/34700363/. Cited 2022 Feb 28. 10.1111/joim.13405 34700363

[26] Siemieniuk RA, Agoritsas T, Macdonald H, Guyatt GH, Brandt L, Vandvik PO (2016). Introduction to BMJ Rapid Recommendations. BMJ.

[27] Bekkering GE, Agoritsas T, Lytvyn L, Heen AF, Feller M, Moutzouri E (2019). Thyroid hormones treatment for subclinical hypothyroidism: a clinical practice guideline. BMJ.

[28] Tikkinen KAO, Dahm P, Lytvyn L, Heen AF, Vernooij RWM, Siemieniuk RAC (2018). Prostate cancer screening with prostate-specific antigen (PSA) test: a clinical practice guideline. BMJ.

[29] Vermandere M, Aertgeerts B, Agoritsas T, Liu C, Burgers J, Merglen A (2018). Antibiotics after incision and drainage for uncomplicated skin abscesses: a clinical practice guideline. BMJ.

[30] Aertgeerts B, Agoritsas T, Siemieniuk RAC, Burgers J, Bekkering GE, Merglen A (2017). Corticosteroids for sore throat: a clinical practice guideline. BMJ.

[31] Siemieniuk RAC, Harris IA, Agoritsas T, Poolman RW, Brignardello-Petersen R, Van de Velde S (2017). Arthroscopic surgery for degenerative knee arthritis and meniscal tears: a clinical practice guideline. BMJ.

[32] van de Velde S, Stichele R vander, Fauquert B, Geens S, Heselmans A, Ramaekers D, et al. EBMPracticeNet: A bilingual national electronic point-of-care project for retrieval of evidence-based clinical guideline information and decision support. JMIR Res Protoc 2013;2(2):e23 https://www.researchprotocols.org/2013/2/e23. 2013;2(2):e2644. Available from: https://www.researchprotocols.org/2013/2/e23. Cited 2022 Feb 10. 10.2196/resprot.2644 PMC3713937 23842038

[33] Eccles DW, Arsal G (2017). The think aloud method: what is it and how do I use it?. Qual Res Sport Exerc Health.

[34] Ericsson KA, Simon HA (1998). How to study thinking in everyday life: contrasting think-aloud protocols with descriptions and explanations of thinking. Mind Cult Act.

[35] ATLAS.ti: The qualitative data analysis & research software. Available from: https://atlasti.com/. Cited 2022 Feb 28.

[36] de DierckxCasterlé B, Gastmans C, Bryon E, Denier Y (2012). QUAGOL: A guide for qualitative data analysis. Int J Nurs Stud.

[37] Rosenbaum SE, Glenton C, Nylund HK, Oxman AD (2010). User testing and stakeholder feedback contributed to the development of understandable and useful summary of findings tables for cochrane reviews. J Clin Epidemiol.

[38] Morville P. User experience design. 2004 https://semanticstudios.com/user_experience_design/. Cited 2021 Apr 27.

[39] Wesolko D. Peter Morville’s user experience honeycomb. 2016. https://medium.com/@danewesolko/peter-morvilles-user-experience- honeycomb-904c383b6886. Cited 2021 Apr 27.

[40] Balshem H, Helfand M, Schünemann HJ, Oxman AD, Kunz R, Brozek J, et al. GRADE guidelines: 3. Rating the quality of evidence. J Clin Epidemiol 2011 64(4):401–6. Available from: https://pubmed.ncbi.nlm.nih.gov/21208779/. Cited 2022 Sep 28. 10.1016/j.jclinepi.2010.07.015 21208779

[41] Muller E, Strukava A, Scholl I, Härter M, Diouf NT, Légaré F, et al. Strategies to evaluate healthcare provider trainings in shared decision-making (SDM): a systematic review of evaluation studies. BMJ Open 2019 9(6):e026488. Available from: https://bmjopen.bmj.com/content/9/6/e026488. Cited 2022 Feb 3. 10.1136/bmjopen-2018-026488 PMC6596948 31230005

[42] Clayman ML, Bylund CL, Chewning B, Makoul G (2016). The impact of patient participation in health decisions within medical encounters. Med Decis Making.

[43] Elwyn G, Frosch DL, Kobrin S (2015). Implementing shared decision-making: consider all the consequences. Implement Sci.

[44] Elwyn G, Frosch D, Thomson R, Joseph-Williams N, Lloyd A, Kinnersley P (2012). Shared decision making: a model for clinical practice. J Gen Intern Med.

[45] Moleman M, Regeer BJ, Schuitmaker-Warnaar TJ. Shared decision-making and the nuances of clinical work: Concepts, barriers and opportunities for a dynamic model. J Eval Clin Pract 2021;27(4):926–34. Available from: https://pubmed.ncbi.nlm.nih.gov/33164316/. Cited 2022 Feb 28. 10.1111/jep.13507 PMC8359199 33164316

[46] Entwistle V, Prior M, Skea ZC, Francis JJ. Involvement in treatment decision-making: its meaning to people with diabetes and implications for conceptualisation. Soc Sci Med. 2008 66(2):362–75. Available from: https://pubmed.ncbi.nlm.nih.gov/17950508/. Cited 2022 Feb 28. 10.1016/j.socscimed.2007.09.001 17950508

[47] Perneger T v., Agoritsas T. Doctors and patients’ susceptibility to framing bias: a randomized trial. J Gen Intern Med. 2011;26(12):1411–7. Available from: https://pubmed.ncbi.nlm.nih.gov/21792695/. Cited 2022 Feb 28. 10.1007/s11606-011-1810-x PMC3235613 21792695

[48] Epstein RM. Communicating evidence for participatory decision making. JAMA 2004 291(19):2359. Available from: http://jama.jamanetwork.com/article.aspx?doi=10.1001/jama.291.19.2359. 10.1001/jama.291.19.2359 15150208

[49] Hersh L, Brooke S, Snyderman D (2015). Health Literacy in Primary Care Practice. Am Fam Physician.

[50] Backonja U, Chi NC, Choi Y, Hall AK, Le T, Kang Y (2016). Visualization approaches to support healthy aging: a systematic review. J Innov Health Inform.

[51] McCaffery KJ, Smith SK, Wolf M (2010). The challenge of shared decision making among patients with lower literacy: A framework for research and development. Med Decis Making.

[52] Durand MA, Carpenter L, Dolan H, Bravo P, Mann M, Bunn F (2014). Do interventions designed to support shared decision-making reduce health inequalities? A systematic review and meta-analysis. PLoS ONE.

[53] Van Weert JCM, Van Munster BC, Sanders R, Spijker R, Hooft L, Jansen J (2016). Decision aids to help older people make health decisions: A systematic review and meta-analysis. BMC Med Inform Decis Mak.

[54] Bunge M, Mühlhauser I, Steckelberg A (2010). What constitutes evidence-based patient information? Overview of discussed criteria. Patient Educ Couns.

[55] Nagel K, Wizowski L, Duckworth J, Cassano J, Hahn SA, Neal M (2008). Using plain language skills to create an educational brochure about sperm banking for adolescent and young adult males with cancer. J Pediatr Oncol Nurs.

[56] Use plain language. Harvard University. Available from: https://accessibility.huit.harvard.edu/use-plain-language. Cited 2021 Dec 21.

[57] Lenert L, Dunlea R, Del Fiol G, Hall LK (2014). A model to support shared decision making in electronic health records systems. Med Decis Making.

[58] Scalia P, Durand MA, Berkowitz JL, Ramesh NP, Faber MJ, Kremer JAM (2019). The impact and utility of encounter patient decision aids: Systematic review, meta-analysis and narrative synthesis. Patient Educ Couns.

[59] Sless D, Shrensky R. Writing about medicines for people. 3rd ed. Sydney: Australian Self-Medication Industry; 2006. Available from: https://www.chpaustralia.com.au/Tenant/C0000022/Documents/Publications/Writing%20About%20Medicines%20for%20People.pdf. Cited 2021 Dec 13.

