# Supplementary material for: Digital encounter decision aids linked to clinical practice guidelines: results from user testing SHARE-IT decision aids in primary care
Source: BMC Med Inform Decis Mak. 2023 May 22;23:97. doi: 10.1186/s12911-023-02186-4 (PMC10201505; doi:10.1186/s12911-023-02186-4)
Supplement: Supplementary file 1 — Additional file 1. Links to EDAs. [file 12911_2023_2186_MOESM1_ESM.docx]

## Additional file 1: Links to EDAs

<https://app.magicapp.org/#/guideline/nyqWPn>

<https://app.magicapp.org/#/guideline/n32gkL>

<https://app.magicapp.org/#/guideline/jlRvQn>

<https://app.magicapp.org/#/guideline/JjXYAL/section/j79pvn>

<https://app.magicapp.org/#/guideline/GnJ7bE>
