# Supplementary material for: Digital encounter decision aids linked to clinical practice guidelines: results from user testing SHARE-IT decision aids in primary care
Source: BMC Med Inform Decis Mak. 2023 May 22;23:97. doi: 10.1186/s12911-023-02186-4 (PMC10201505; doi:10.1186/s12911-023-02186-4)
Supplement: Supplementary file 2 — Additional file 2. Semi-structured interview questions. [file 12911_2023_2186_MOESM2_ESM.docx]

## Additional file 2: Semi-structured interview questions

Your general practitioner discussed some information with you concerning your treatment option on a tablet / pc.

1. What did you think about this information?
2. Was it clear, easy to understand?
3. Did you like the way it was displayed?
4. Tell me about some good and/or bad things.
5. What could be shown better/different?
6. How do you feel about your general practitioner using this tool?
7. Do you think it helped you and your general practitioner making a decision today?
